# Gut microbiome diversity and biogeography for Chinese bumblebee *Bombus pyrosoma*

**DOI:** 10.1128/msystems.00459-24

**Published:** 2024-06-27

**Authors:** Zhengyi Zhang, Yulong Guo, Mingsheng Zhuang, Fugang Liu, Zhongyan Xia, Zhihao Zhang, Fan Yang, Huayan Zeng, Yueguo Wu, Jiaxing Huang, Kai Xu, Jilian Li

**Affiliations:** 1 State Key Laboratory of Resource Insects, Institute of Apicultural Research, Chinese Academy of Agricultural Sciences, Beijing, China; 2 Luoping Yunling Bee Industry and Trade Co., Ltd, Yunnan, China; 3 Apiculture science Institute of Jilin Province, Jilin, China; Third Institute of Oceanography Ministry of Natural Resources, Xiamen, China

**Keywords:** gut microbiota, *Bombus pyrosoma*, community assembly, stochastic drift

## Abstract

Gut microbiota of the bumblebee is critical as it modulates the health and fitness of the host. However, the mechanisms underlying the formation and maintenance of the diversity of bumblebee gut bacteria over a long period of evolution have yet to be elucidated. In particular, the gut bacterial diversity and community assembly processes of *Bombus pyrosoma* across the Chinese border remain unclear. In this study, we systematically carried out unprecedented sampling of 513 workers of the species *Bombus pyrosoma* across the Chinese landscape and used full-length 16S rRNA gene sequencing to examine their gut microbiota diversity and biogeography. The gut microbiota composition and community structure of *Bombus pyrosoma* from different geographical locations were diverse. On the whole, the gut bacteria *Gilliamella* and *Snodgrassella* are dominant in bumblebees, but opportunistic pathogens *Serratia* and *Pseudomonas* are dominant in some sampling sites such as Hb15, Gs1, Gs45, Qhs15, and Ssx35. All or part of environmental factors such as latitude, annual mean temperature, elevation, human footprint, population density, and annual precipitation can affect the alpha diversity and community structure of gut bacteria. Further analysis showed that the assembly and shift of bumblebee gut bacterial communities under geographical variation were mainly driven by the stochastic drift of the neutral process rather than by variable selection of niche differentiation. In conclusion, our unprecedented sampling uncovers bumblebee gut microbiome diversity and shifts over evolutionary time.

**IMPORTANCE:**

The microbiotas associated with organisms facilitates host health and fitness, and the homeostasis status of gut microbiota also reflects the habitat security faced by the host. In addition, managing gut microbiota is important to improve bumblebee health by understanding the ecological process of the gut microbiome. Thus, we first carried out an runprecedented sampling of 513 workers of the species *Bombus pyrosoma* across the Chinese landscape and used full-length 16S rRNA gene sequencing to uncover their gut microbiota diversity and biogeography. Our study provides new insights into the understanding of gut microbiome diversity and shifts for Chinese Bumblebee over evolutionary time.

## INTRODUCTION

Bumblebees and honeybees are important insects through which pollination improves crop yield and quality and plays an important role in maintaining plant diversity and stability of local ecosystems (1). Bumblebees have a large body and thick body hair, which can accommodate more pollen and better contact with flowers (2). In addition, bumblebees are good at buzz pollination, and their unique pollination behavior makes them efficient pollinators (3). However, there is a global decline of many wild bumblebee species populations, probably owing to these insects facing multiple threats such as the adverse impacts of toxins or poor nutrition and disturbances in gut microbiota (4). Therefore, maintaining gut homeostasis is a key factor for bumblebee conservation because gut microbes can regulate host health and fitness by degrading toxins, resisting pathogen invasion, and promoting nutrient absorption (5, 6).

Recent research has revealed that naturally occurring microbiomes of bumblebees have evolved over time with hosts and positively impact host health (7). In general, gut microbiota of bumblebees consist of at least five core genera: *Gilliamella*, *Snodgrassella*, *Bifidobacterium*, *Lactobacillus*, and *Bombilactobacillus* (8). Several members of the core genera have been found in bumblebees, such as *Schmidhempelia* and *Bombiscardovia* (7). Native gut microbiota maintain the health of bee colonies through their involvement in various processes, including protection against pathogens, digestion, detoxification of food, development, and immune stimulation (9). However, for bumblebees, like humans, disorder in the gut microbiome is a key factor for the onset of many local and systemic diseases (10), and gut microbiota also exhibits a community composition of the dominant environmental pathogenic bacteria whenin a disorder (11). The aforementioned research suggests that homeostasis or disturbance of the gut microbiome reflects the ecological status of the host. Therefore, understanding the gut composition in different habitats is necessary for the perspective of bumblebee ecological conservation.

Microbiota has an essential role in the health and development of their animal hosts (12). Transmission of gut bacteria is also critical to the host, which can enable governing of bumblebee gut bacterial communities for better managing gut microbiota for improving bumblebee health and fitness. Many factors affect the honeybee gut bacterial community, including host attributes (e.g., species, age, and caste), external environment (e.g., diet and antibiotics), and possibly geographic locations (9, 13). Studies have shown that mean annual temperature and mean annual precipitation in different geographical locations can significantly affect the diversity of gut bacteria in the honeybees of the same species (7, 14). Furthermore, ecological processes drive the convergence (e.g., homogeneous selection and variable selection) and divergence (e.g., homogeneous dispersal, dispersal limitation, and stochastic drift) of gut microbes (15–17). Studies have shown that geographical variation of honeybee gut bacterial communities is mainly driven by an undominated process (14). Such studies have initiated new insights into the factors that influence honeybee gut bacterial communities. However, bumblebees have a different host ecology and gut-specific microbiota compared with honeybees. Moreover, to date, the processes that drive the assembly and shift of gut bacterial communities in wild bumblebees over evolutionary time have remained elusive, especially under different geographical conditions.

China possesses rich species of wild bumblebees, such as *Bombus pyrosoma*, which contains a simple yet specialized gut microbiota and spans different ecosystems from low elevations in the North China Plain to high elevations in the Qinghai–Tibet Plateau (18, 19). Thus, this bumblebee species provides an ideal system to study gut microbial composition and mechanisms of microbial species coexistence. In this study, we conducted methodical sampling of gut microbiota in 513 workers of *Bombus pyrosoma* from 58 locations of nine provinces across the Chinese landscape and further performed full-length 16S rRNA gene sequencing. This work enables us to know the diversity and shifts of the bumblebee gut microbiome under geographical variation. Bumblebees in some areas such as Gansu province have a gut composition of dominant environmental pathogenic bacteria such as *Pseudomonas*. External factors influence the diversity and community structure of gut microbes. In addition, the assembly and shift of bumblebee gut bacterial communities under geographical variation was mainly driven by stochastic drift rather than variable selection. These findings provide important insights into the bumblebee gut microbiome diversity, biogeography, and host protection.

## MATERIALS AND METHODS

### *Bombus pyrosoma* sample collection and DNA extraction

**Fig 1:**
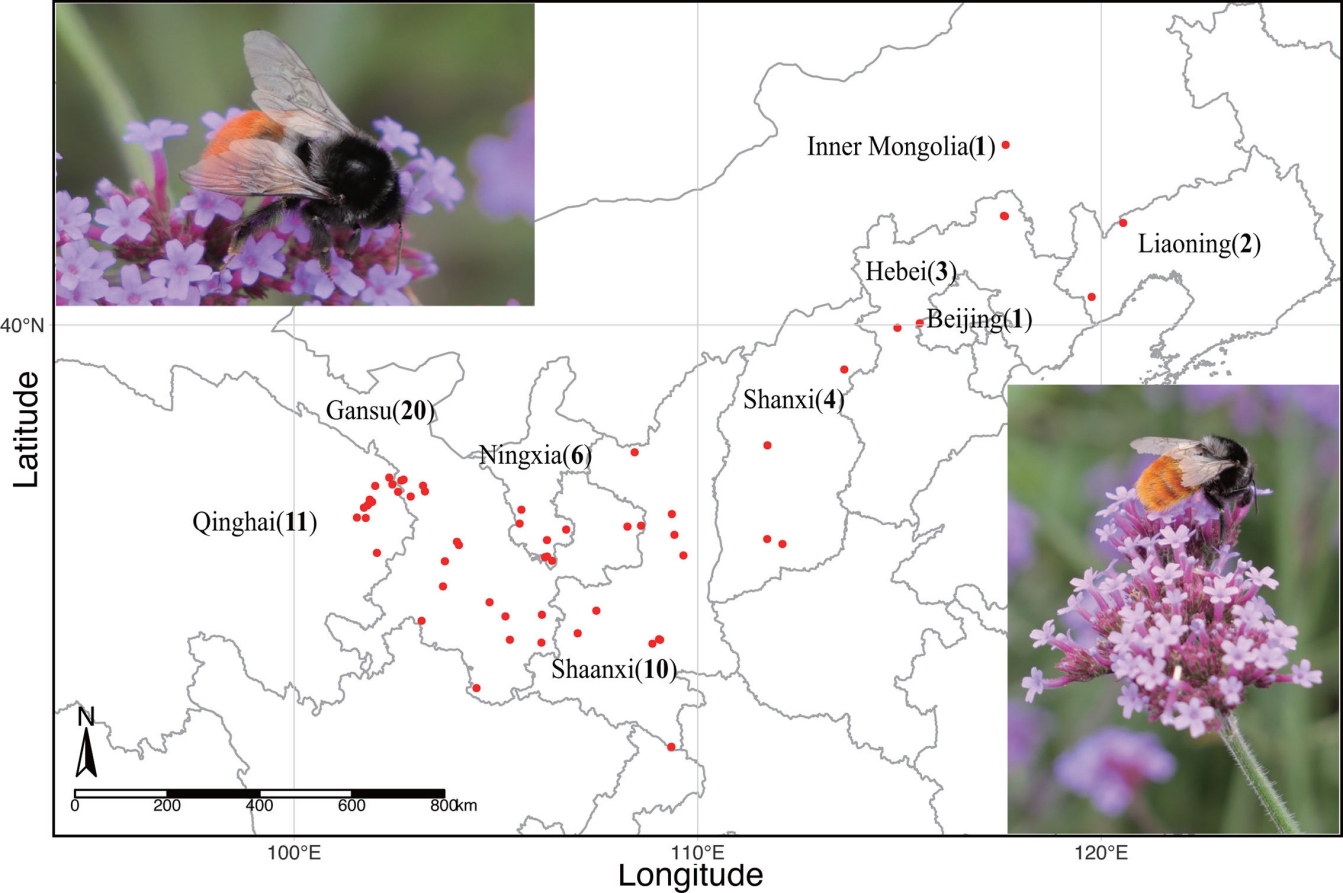
The map of Mainland China showing the *Bombus pyrosoma* sampling sites in this study. A total of 513 workers of *Bombus pyrosoma* were collected from 58 different sites (red dots) in nine provinces; bold numbers represent the sampling points in each province. There were 3–20 biological replicates per sampling site. The map was created with R (v 4.1.1).

We collected 513 individual bumblebee workers of the same species (*Bombus pyrosoma*) from 58 different sites across nine provinces in China (Fig. 1; Table S1). The gut DNA of the bumblebees was extracted according to the protocol of the Wizard Genomic DNA Purification Kit (A1125; Promega, Madison, WI, USA). The extracted DNA was dissolved in 50 uL of Tris-EDTA (TE) buffer, quantified by a NanoDrop 2000 ultraviolet–visible (UV-vis) spectrophotometer (NanoDrop, DE, USA), and qualitatively evaluated by gel electrophoresis.

### Sequencing and data analysis

PCR amplification and DNA library construction associated with full-length 16S rRNA gene sequencing were conducted according to previously published methods (20). Briefly, full-length bacterial 16S rRNA gene sequencing was conducted using the forward primer 27F (5′-AGRGTTYGATYMTGGCTCAG-3′) and the reverse primer 1492R (5′-RGYTACCTTGTTACGACTT-3′). The PCR reaction conditions were 95°C for 5 min, 95°C for 30 s, 50°C for 30 s, and 72°C for 1 minute, with 25 cycles in a reaction volume of 10 µL, and PCR amplification was performed using the PacBio Sequel platform at Beijing Personal Biotechnology Co., Ltd. (Beijing, China). The following analyses were performed on the Qiime2 (v 2023.2.0) platform (21). The commands “qiime dada2 denoise-ccs” and “qiime taxa filter-table” were used to obtain amplicon sequence variants (ASVs) associated with filtering these features (mitochondrial and chloroplast reads, eukaryotic reads, and chimeric reads) and feature table. The obtained ASVs were taxonomically annotated in the SILVA 138 database with the command “qiime feature-classifier” (22). Alpha and beta diversity were calculated using the command “qiime diversity core-metrics-phylogenetic --i-phylogeny rooted-tree.qza --i-table table.qza --p-sampling-depth 4530 --m-metadata-file sample-metadata.tsv --output-dir core-metrics-result,” and “--p-sampling-depth 4530” and “--p-sampling-depth 4530” mean that all samples were rarefied 4,530 sequences (4,530 sequences per sample).

### Enterotype analyses

The sample set was examined for the presence of enterotypes using previously described methods (23). Briefly, a Jensen–Shannon distance matrix was generated and used to create clusters with the partitioning around medoid (PAM) method. The optimal cluster number was evaluated via the Calinski–Harabasz (CH) index, with validation of cluster strength by the Silhouette method. Clusters were visualized in a principal coordinate analysis (PCA) plot. These techniques and the R scripts used to perform them are available on the web tutorial located at http://enterotype.embl.de/enterotypes.html.

### Environmental variables

Environmental variables of annual mean temperature and annual precipitation were the standard WorldClim Bioclimatic variables downloaded from WorldClim 2.1 at a 2.5 min resolution with the average for the years 1970–2000 (Table S1) (24). The elevation variables were downloaded from WorldClim 2.1 at a 2.5 min resolution and were derived from the shuttle radar topography mission (SRTM) elevation data (Table S1) (24). The human footprint and population density were developed by Sanderson and colleagues (25), which are mainly used to reflect the indicators of human interference. (25, 26) (Table S1).

We screened environmental factors to satisfy the subsequent analysis of the association between environmental factors and gut bacterial diversity. First, the correlation between the six environmental variables (e.g., latitude, elevation, annual mean temperature, annual precipitation, population density, and human footprint) and ASV-feature table is significant (*P* < 0.05) through the adonis function (Table S2). Second, we conduct collinearity analysis on the six environmental variables through the vif.cca function (27), and the variance inflation factor of each environmental variables is less than 10 (Table S2), which address the effects of strong collinearity of environmental factors.

### Division of ecological processes

The estimation of five ecological processes followed previously described methods. Stegen et al. proposed a method called “quantifying community assembly processes and identifying features that impose them” (28, 29). Initially, we calculated both β-nearest taxon index (βNTI) and Raup–Crick index (RC) using null models with 1,000 randomizations. Subsequently, we integrated βNTI and RC to determine the relative dominance of the following factors in shaping the composition of the gut bacterial community: homogeneous selection (βNTI < −2), variable selection (βNTI > 2), homogeneous dispersal (RC >0.95 and |βNTI| < 2), dispersal limitation (RC >0.95 and |βNTI| < 2), and stochastic drift (|RC| < 0.95 and |βNTI| < 2) (Table S3). These five ecological processes that can lead to community convergence (homogenous selection and homogeneous dispersal) and divergence (variable selection, dispersal limitation, and stochastic drift) (14).

### Statistical analysis

All statistical analyses were performed on the R (v 4.1.1) platform. Mean relative abundances of genus level between two enterotypes were tested for statistical difference using Kruskal–Wallis tests with kruskal.test function (30). Pairwise permutation multivariate analysis of variance (PERMANOVA) with 999 random permutations was employed to test the significance of the differences among the samples of different provinces and sites using the vegan package (31). Linear fitting of external factors and alpha diversity (e.g., chao1 and faith_pd) used glm function from generalized linear models because of the presence of Poisson distribution (Fig. S1) (32). Distance-decay relationship (DDR) analysis used the geosphere package (33). Canonical correspondence analysis (CCA) based on Bray–Curtis distance was visualized with the ggplot2 package (34) and the ANOVA test was carried out with the permutest function (35). The envfit function was used to test the correlation between each environmental factor and gut community (36). The prop.test function was conducted to test whether the geographical site induced changes in the relative importance of ecological processes (37).

## RESULTS

### Microbial community diversity of *Bombus pyrosoma* under geographical variation

A total of 15,810,688 sequences from 513 samples (30,820 reads per sample) generated 1,937 ASVs (Table S4 and S5). The accumulation curve based on community observed_features showed that diversity reached saturation with the increase in sequencing depth (Table S6). For the alpha diversity of microbial communities, the chao1 index of the 513 gut samples from 58 different regions ranged from 4 to 129, with an average of 18.52 (Fig. S2; Table S7). Faith_pd (Faith’s Phylogenetic Diversity) based on phylogeny ranged from 2.61 to 6.14, with an average of 3.31 (Fig. S2; Table S7). Regarding the beta diversity based on Bray–Curtisdistance of microbial communities, the PERMANOVA showed that the gut bacteria are significantly differentiated by provinces (*P* = 0.001) and sites (*P* = 0.001) (Fig. S3).

### Taxonomy of gut microbiota

**Fig 2:**
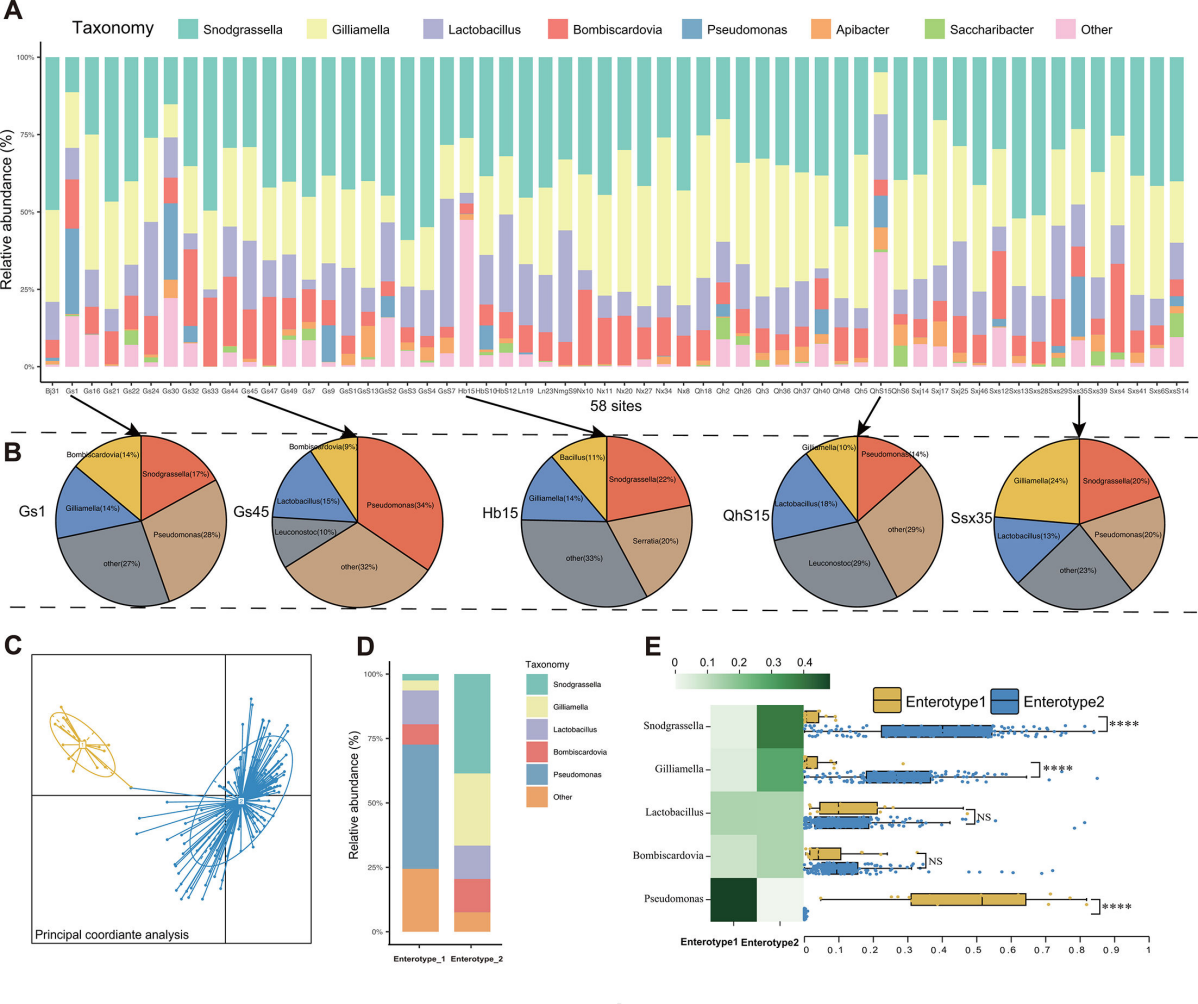
Gut microbiota taxonomy of *Bombus pyrosoma* from different locations in China (samples, *n* = 513). (**A**) Relative abundance at the genus level. (**B**) Relative abundance at the genus level in areas of pathogenic bacteria dominance. (**C**) Principal component analysis (PCA) plot of enterotype clusters observed in Gansu province. (**D**) Mean relative abundances at the genus level between enterotype 1 and enterotype 2. (**E**) Heat map showing mean relative abundance of genera for both enterotypes, and boxplots showing differences (Kruskal–Wallis test) in mean relative abundance of bacterial genera between the two enterotypes. ****, *P* < 0.0001; NS, not significant, (*P* > 0.05).

Overall, based on the relative abundance, the top four gut bacteria from 513 individual worker bees in 58 regions were the previously reported core symbiotic bacteria *Gilliamella*, *Snodgrassella*, *Lactobacillus*, and *Bombiscardovia*. The genera *Pseudomonas*, *Apibacter*, *Saccharibacter*, and others typically appear in the bumblebee gut as noncore bacteria (Fig. 2A; Table S5). However, *Pseudomonas* appeared in sampling sites such as Gs1 (28% *Pseudomonas*) and Gs30 (34% *Pseudomonas*) in Gansu province, Qhs15 (14% *Pseudomonas*) of Qinghai province, and the Sxs35 (20% *Pseudomonas*) of Shaanxi province, replacing the dominant positions of *Gilliamella* and *Snodgrassella* (Fig. 2B). Even in some areas of Hebei province (Hb15), a community structure similar to the previously studied enterotype was found, with a higher proportion of potentially pathogenic bacteria (20% *Serratia*) (Fig. 2B).

Owing to the presence of numerous opportunistic pathogens in individual bumblebees in Gansu province, we conducted optimal clustering using the CH index and the Silhouette index based on the PAM algorithm. PCA revealed the existence of two enterotypes within the 160 samples collected from Gansu province (Fig. 2C; Table S8). Among these, enterotype 2 (*n* = 144) exhibited similarities with previously identified enterotypes dominated by *Gilliamella* and *Snodgrassella*; conversely, enterotype 1 (*n* = 16) was characterized by a dominance of *Pseudomonas* (Fig. 2D; Table S8). By comparing the relative abundance of the two enterotypes of gut bacteria, the core native gut bacteria of enterotype 1 exhibited a significant reduction, whereas the presence of the pathogenic bacterium *Pseudomonas* substantially increased as the dominant bacterial group (Fig. 2E; Table S8). The relative abundances of *Lactobacillus* and *Bombiscardovia* in the two enterotypes showed no statistically significant differences (Fig. 2E; Table S8).

### Correlation between external factors and community diversity

**Fig 3:**
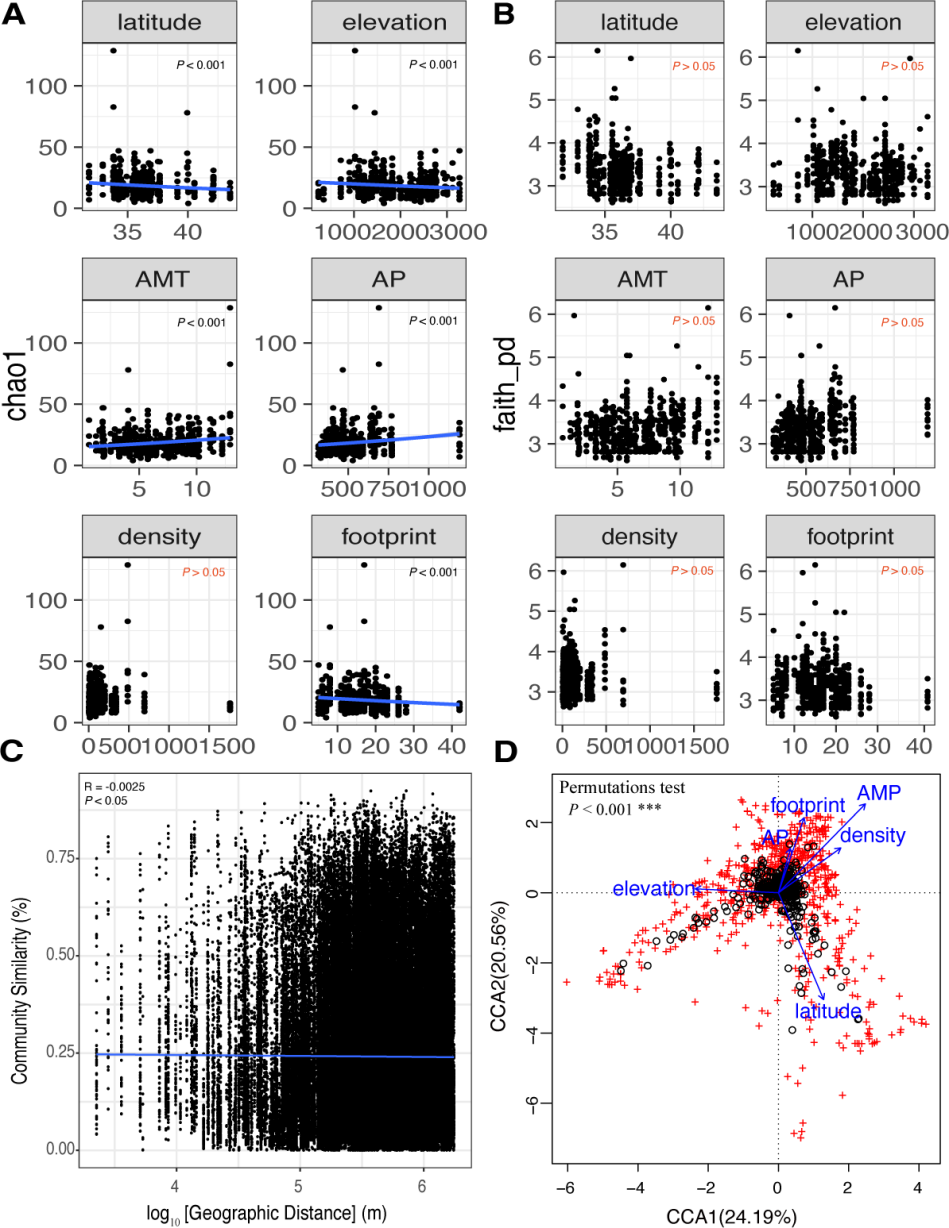
Correlation between external factors and community diversity. (**A**) Generalized linear fitting of the chao1 index of alpha diversity and environmental factors; the red marks are not significant. (**B**) Generalized linear fitting of faith_pd index of alpha diversity and environmental factors; the red marks are not significant. (**C**) Distance-decay relationships (DDRs) based on Bray–Curtis dissimilarity of gut community, *P* < 0.05. (**D**) Canonical correspondence analysis (CCA) showed that the arrow length represents the strength of the correlation between the environmental variables and the microbes. The longer the arrow length, the stronger the correlation. The perpendicular distance between microbes and environmental variable axes in the plot reflects their correlations. The smaller the distance, the stronger the correlation. AMT, annual mean temperature; AP, annual precipitation.

Regarding the alpha diversity based on chao1 of gut microbiota, five external factors (latitude, elevation, annual mean temperature, annual precipitation and human footprint) showed statistically significant linear relationships (*P* < 0.001) (Fig. 3A), although the population density did not have a significant linear relationship with the alpha diversity based on chao1. As latitude, elevation, and human footprint increased, the chao1 indices of gut microbes decreased (Fig. 3A). However, with the increase in annual mean temperature and annual precipitation, the alpha diversity of gut microbiota in bumblebees increased (Fig. 3A). Six external factors (latitude, elevation, annual mean temperature, annual precipitation, population density, and human footprint) did not exhibit a statistically significant linear relationship with the alpha diversity based on faith_pd of gut microbiota (Fig. 3B). The detailed statistical results based on the generalized linear model are shown in Table S9.

In addition to alpha diversity, we explored the relationship between microbial community similarity and geographic distance based on the Bray–Curtis distance. The similarity of the gut microbial communities of bumblebees varies with increasing geographic distance (*P* < 0.05), although the linear correlation coefficient was low (Fig. 3C). Canonical correspondence analysis showed that the annual mean temperature had the greatest correlation with gut microbial composition, whereas annual precipitation had the weakest correlation (Fig. S4). Overall, six variables—latitude, elevation, annual mean temperature, annual precipitation, population density, and human footprint—presented a significant correlation with microbial community composition (*P* < 0.001) (Fig. S4).

### The relative contribution of community assembly processes of *Bombus pyrosoma*

**Fig 4:**
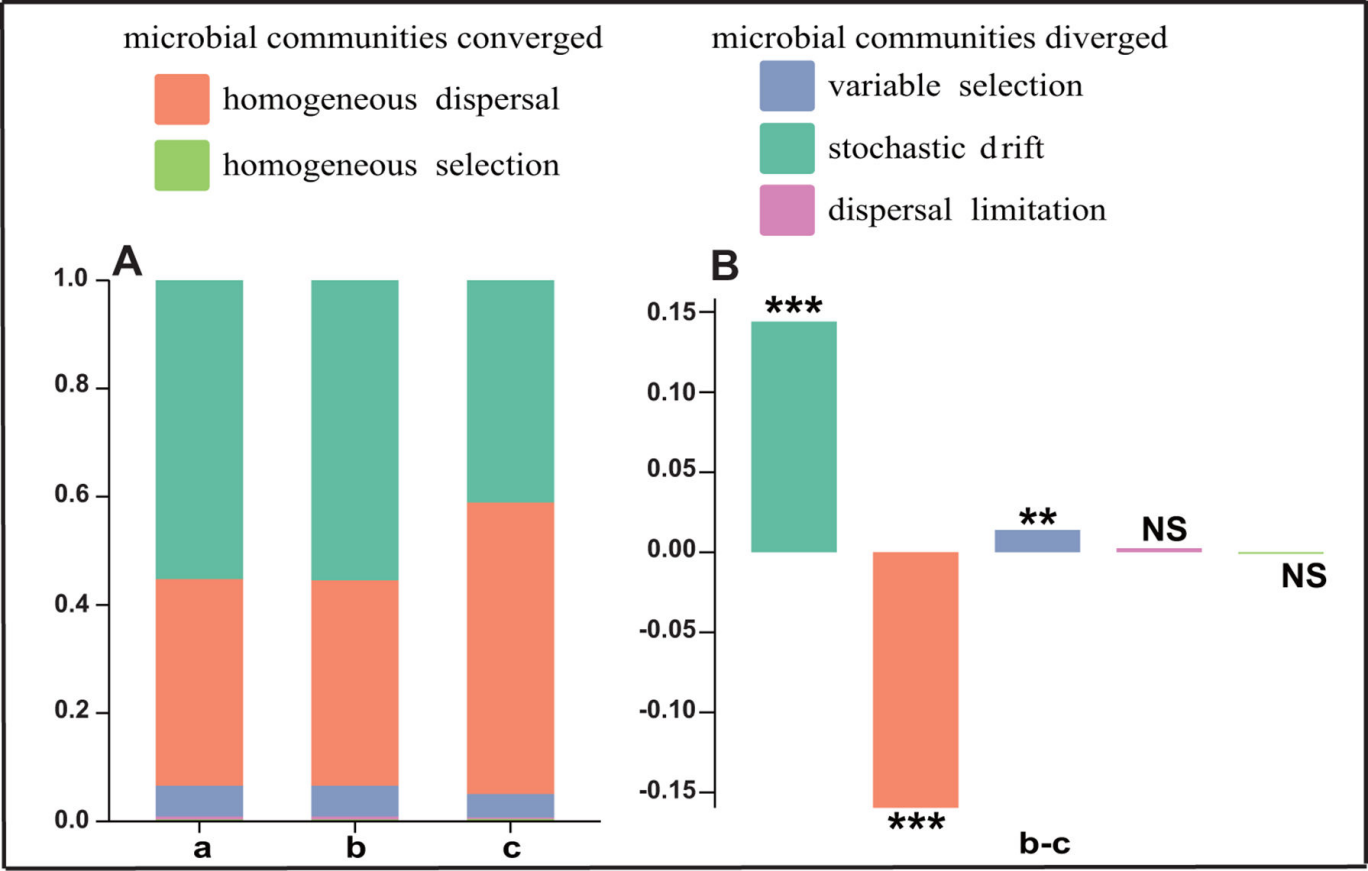
Ecological processes driving the assembly and shift of bumblebee gut bacterial communities. (**A**) The relative importance of ecological processes in governing bumblebee gut bacterial communities under three conditions: without considering geographical location (“a”), different geographical locations (“b”), and the same geographical locations (“c”). (**B**) “b–c” represents site-induced changes in the relative importance of ecological processes in governing the shift of bumblebee gut bacterial communities. ***, *P* < 0.001; **, *P* < 0.01; NS, not significant (*P* > 0.05).

Without considering the influence of geographical location, the relative contribution of the signal driving the change of gut microbial community was 55.19% stochastic drift, 38.28% homogeneous dispersal, 5.20% variable selection, 0.52% dispersal limitation, and 0.36% homogeneous selection (Fig. 4A). The relative contribution of the gut microbial community assembly processes of bumblebees in the same geographical location was 41.06% stochastic drift, 53.89% homogeneous dispersal, 4.33% variable selection, 0.28%, dispersal restriction, and 0.44% homogeneous selection (Fig. 4A). Stochastic drift of 55.46%, homogeneous dispersal of 37.93%, variable selection of 5.73%, dispersal limitation of 0.52%, and homogeneous selection of 0.36% were the relative contributions of community assembly processes in 58 different geographical locations (Fig. 4A).

Further analyses showed that the intervention of different geographical locations caused changes in the relative contribution of ecological processes (Fig. 4B). The homogeneous dispersal process of gut bacterial communities was significantly reduced (*P* < 0.05), whereas the stochastic drift and variable selection ecological processes, which lead to the divergence of gut bacterial communities, were significantly increased (*P* < 0.05) (Fig. 4B). Stochastic drift had a much larger effect than variable selection and thus is a major driving mode for gut microbiome divergence (Fig. 4B).

## DISCUSSION

This is the first study conducting an unprecedented investigation into the composition and diversity of gut microbiota in the worker bees of Chinese native bumblebees (*Bombus pyrosoma*), covering 513 samples from almost all the distributed ranges of this species. Our massive PacBio full-length sequencing effort revealed the presence of 1,937 microbial ASVs (Table S5), which may represent the overall microbial diversity of *Bombus pyrosoma*. In addition, our results show that alpha diversity (e.g., chao1 and faith_pd) of individual gut microbiota is diverse across different regions and even in some of the same regions (Fig. S2; Table S7). Beta diversity is also significantly differentiated with varying groups (e.g., provinces and sites) (Fig. S3). These results reinforce those of earlier research, suggesting that host sociality facilitates the development and maintenance of specialized microbiomes (7). Despite the same hosts sharing similar social behavior, the complexity of gut microbiota diversity arises owing to the vertical transmission from queen bees shaping the offspring’s gut composition, with an additional contribution of horizontal transmission through social interactions and environmental exposure (38). These intricate factors could result in varying alpha diversities—even within the same host of the same geographical location—and varying beta diversities from different groups of geographical locations and provinces.

Our survey of the gut microbiome under landscape found that some sampling sites (e.g., Gs1, Gs30, Qhs15, Sxs35, and Hb15) had a gut composition of dominant environmental pathogenic bacteria. This was particularly observed in Gansu province where an enterotype characterized by the predominance of *Pseudomonas* has emerged (Fig. 2E), suggesting bumblebees may be suffering from severe external pressure and coercion. The composition and community structure of gut microbiota have always been an early warning system for host health and fitness (6). An imbalance in gut homeostasis may arise from external disturbances to the host’s environment, such as environmental pollution and antibiotic misuse, leading to the intrusion of certain exogenous pathogenic conditions into the host (39). For humans, such disruption of protective barriers can lead to abnormal immune responses to the microbial community and is a hallmark of various chronic inflammatory diseases, including inflammatory bowel disease, HIV/AIDS, viral hepatitis, cardiovascular diseases, and cancers, posing significant health risks to the host (40). Previous research has shown that the enterotype characterized by *Hafnia* and *Serratia* can invade the host’s immune system (11). However, unlike *Hafnia* and *Serratia*, *Pseudomonas* is a globally ubiquitous bacterium, including species such as *P. aeruginosa*, *P. oryzihabitans*, and *P. syringae*, with significant pathogenicity for animals and plants (41, 42). In humans, *Pseudomonas* infection can cause acute infections and chronic lung infections (43). Therefore, the gut microbiota of wild Chinese bumblebees *Bombus pyrosoma* may harbor pathogens that could severely affect the health of hosts in Gansu province. The reasons underlying this predominance of pathogenic bacteria may be environmental pollution and the spread of disease caused by the dominant animal husbandry in this region, which highlights the urgent need to create a favorable habitat for bumblebees. The disruption of gut microbiota in some individuals from different sites may also be linked to bee colony age, as studies have shown that highest prevalence in *B. atratus* sampled later in the colony cycle (44, 45).

Numerous factors influence the shaping of gut microbiota, and although host species remain a primary determinant, the influence of external factors on bumblebee gut microbiota cannot be ignored (13, 46). We found that alpha diversity of gut microbiota decreased significantly with increasing latitude (Fig. 3A), demonstrating that bumblebee gut bacteria have a latitudinal diversity gradient, which is one of the broadest and most important patterns of biodiversity on Earth (47). The alpha diversity of gut microbiota increased with rising average annual mean temperature and annual precipitation (Fig. 3A). As honey source plants are usually found in low latitude, high temperature, and humid places, the bumblebees associated with their feeding have more diversity of gut bacteria. Considering the potential impact of human activities on bumblebee habitats, we discovered that as the human footprint increased, the alpha diversity of gut microbiota significantly decreased (Fig. 3A). It is conceivable that the human footprint might affect a decrease in the number of bumblebee queens and colonies, leading to a loss of gut microbial diversity. We also found that six external factors were significantly correlated with gut microbial community structure (Fig. 3C and D). In summary, the external environment can shape the alpha and beta diversity of gut microbes.

Studies of community assembly processes are crucial for explaining species coexistence and the maintenance of biodiversity (48). By analyzing five ecological processes, we explored the relative contribution of gut microbial community assembly processes in bumblebees and how these relative contributions vary among different geographical locations. Without considering geographical location, despite variable selection (5.20%) and dispersal limitation (0.52%) (Fig. 4A), we found that stochastic drift (55.19%) was a major driver of gut microbial community assembly. This stochastic drift results in unpredictable fluctuations in microbial communities, leading to heterogeneity, even within the gut microbial structures of individuals from the same region. The dominant force for convergence of community structures was homogenizing dispersal (38.28%) rather than homogenizing selection (0.36%) (Fig. 4A), and as microbial dispersal is constrained by similar physical barriers after microbial colonization in the guts of hosts of the same species, this results in a similar community of microbes. Considering the effect of geographical location, based on microbial community assembly and shift from the same region to different regions, further expansion of stochastic drift drives variation and uncertainty in the microbial community structure within and between groups (Fig. 4B). Different geographical locations have different ecological environments (e.g., annual mean temperature and annual precipitation), where the diversity of plants associated with host foraging is subject to environmental selection, thus providing more opportunities for the diversity of bumblebee gut microbes. For example, gut microbial diversity was significantly correlated with external factors such as elevation, as mentioned above, which indicates that gut microbes are driven by variable selection. However, microbial community divergence was mainly driven by stochastic drift rather than variable selection under geographical variation (Fig. 4B). Dispersal restriction results in limited active diffusion ability of microorganisms colonized in the same gut (Fig. 4B), while the same host exhibits homologous filtration behavior, maintaining homogenization of the same gut microbiota. Different environmental filtering due to geographical locations indirectly weakens the contribution of dispersal limitation. Briefly, geographical isolation facilitates stochastic processes to shape both host genetic diversification and random changes in gut microbiota (49, 50), processes further influenced by geographical factors such as local environment, latitude, MAT, and MAP (51) . In the comprehensive coevolutionary history of bumblebee gut bacterial communities, neutrality-based stochastic processes emerge as the primary driving forces, while deterministic processes dictate the trajectory of coevolution.

Although our work relies on relative abundance assessment to determine the heritability of the bumblebee gut microbiome, it is important to acknowledge its limitations. Typically, microbiome heritability is inferred from relative abundance data, where taxon-specific abundances are expressed as proportions of the total microbial abundance in a sample. However, research by Marjolein *et al*. had revealed that relying solely on relative abundance data may misrepresent the heritability of the microbiome (52). Three key issues arise when using relative abundances to estimate microbiome heritability: (1) the interdependency between taxa can lead to imprecise heritability estimates (2). Large sample size leads to high false discovery rates (3). Microbial co-abundances lead to biased heritability estimates (52). As such, it is imperative for future investigations to incorporate absolute abundance measurements to provide a more comprehensive understanding of microbiome heritability.

### Conclusions

We collected 513 workers of bumblebee *Bombus pyrosoma* at 58 sites across the Chinese landscape. Bumblebees in some areas had a gut composition of dominant environmental pathogenic bacteria, especially in Gansu province. External factors affect diversity and community structure of gut microbes. In addition, the assembly and shift of bumblebee gut bacterial communities under geographical variation was mainly driven by stochastic drift rather than variable selection. Overall, our unprecedented sampling effort of bumblebee gut microbiomes provides new insights into the microbial diversity, the factors driving their distribution, and understanding ecological processes of gut microbiota.

## Data Availability

The PacBio circular consensus sequencing (CCS) reads have been deposited in the National Center for Biotechnology Information (NCBI) Sequence Read Archive (SRA) with the accession number PRJNA1027760.

## AFTER EPUB

*[This article was published on 27 June 2024 with an incorrect accession number in the Data Availability statement. The accession number was corrected in the current version, posted on 8 July 2024.]*

## SUPPLEMENTAL MATERIAL

Fig. S1 msystems.00459-24-s0001.eps Distribution histogram of Chao1 and Faith_pd.

Fig. S2 msystems.00459-24-s0002.eps Alpha diversity based on Chao1 and Faith_pd of *Bombus pyrosoma* at 58 different sites.

Fig. S3 msystems.00459-24-s0003.eps Alpha diversity based on Chao1 and Faith_pd of *Bombus pyrosoma* at 58 different sites.

Fig. S4 msystems.00459-24-s0004.eps Correlation between six environmental factors and gut microbial communities.

Legends msystems.00459-24-s0005.docx Legends for Fig. S1 to S4.

Supplemental tables msystems.00459-24-s0006.xlsx Tables S1 to S9.

ASM does not own the copyrights to Supplemental Material that may be linked to, or accessed through, an article. The authors have granted ASM a non-exclusive, world-wide license to publish the Supplemental Material files. Please contact the corresponding author directly for reuse.
