# Supplementary material for: Gut microbiome diversity and biogeography for Chinese bumblebee Bombus pyrosoma
Source: mSystems. 2024 Jun 27;9(7):e00459-24. doi: 10.1128/msystems.00459-24 (PMC11264632; doi:10.1128/msystems.00459-24)
Supplement: Legends — for Fig. S1 to S4. [file msystems.00459-24-s0005.docx]

Supplementary Material

Gut microbiome diversity and biogeography for Chinese wild Bumblebee *Bombus pyrosoma*

Zhengyi Zhang^1^, Yulong Guo^1^, Mingsheng Zhuang^1^, Fugang Liu^1^, Zhongyan Xia^1^, Zhihao Zhang^1^, Fan Yang^1^, Huayan Zeng ^2^, Yueguo Wu^2^, Jiaxing Huang^1^, Kai Xu^3^*, Jilian Li^1^*

^1^ State Key Laboratory of Resource Insects, Institute of Apicultural Research, Chinese Academy of Agricultural Sciences, Beijing, 100093, China

^2^ Luoping Yunling Bee Industry and Trade Co., Ltd, Yunnan, 655899, China

^3^Apiculture science Institute of Jilin Province, Jilin, 132108, China

*** Correspondence:**Kai Xu
[xukaiyuzhong@126.com](mailto:xukaiyuzhong@126.com)

Jilian Li
[bumblebeeljl@hotmail.com](mailto:bumblebeeljl@hotmail.com)

**Figure S1.** Distribution histogram of Chao1 (the left) and Faith_pd (the right), which were biased towards the poisson distribution.

**Figure S2.** The alpha diversity based on Chao1 (A) and Faith_pd (B) of *Bombus pyrosoma* at 58 different sites.

**Figure S3.** The PCOA based on Bray–Curtis distances depicting the gut bacterial community composition among provinces (the left) and sites (the right), the PERMANOVA was applied to detect the differences in different group.

**Figure S4.** The correlation between six environmental factors and gut microbial communities. ***, *P* < 0.001.
